# Effect of the daily duration of calf contact on the dam's ultradian and circadian activity rhythms

**DOI:** 10.3168/jdsc.2023-0465

**Published:** 2024-03-02

**Authors:** Marie Schneider, Christina Umstätter, Hassan-Roland Nasser, Eva Gallmann, Kerstin Barth

**Affiliations:** 1 Johann Heinrich von Thünen Institute, Federal Research Institute for Rural Areas Forestry and Fisheries, Institute of Organic Farming, 23847 Westerau, Germany; 2 University of Hohenheim, Center for Livestock Technology, Garbenstraße 9, 70599 Stuttgart, Germany; 3 Johann Heinrich von Thünen Institute, Federal Research Institute for Rural Areas Forestry and Fisheries, Institute of Agricultural Technology, 38116 Braunschweig, Germany; 4 Agroscope, Digital Production, Tänikon, 8356 Ettenhausen, Switzerland

## Abstract

Cow-calf contact systems are attracting increasing interest among farmers and some are already being implemented into dairy farms. However, a comprehensive assessment of animal welfare in these systems is lacking. One reason for this is the large amount of time required for behavioral observations. However, the increased use of sensors in herd management assistance systems offers new opportunities for automated monitoring of animal welfare. For example, accelerometers can be used to collect activity data for a specific pattern analysis. In this study, ultradian and circadian rhythms of cows were analyzed. The degree of functional coupling (DFC; range of values: 0–1) expresses the extent to which the activity is cyclic to 24 h, and therefore harmonically synchronized with the periodicity of the environment. A DFC of 1 indicates complete adaptation of the cows' activity rhythm to the 24-h day. Additionally, the diurnality index (DI) is used to examine the distribution of diurnal and nocturnal activity. A DI of 1 indicates complete diurnal activity, whereas −1 indicates complete nocturnal activity. The rhythms of healthy and well-adapted animals show high adaptation to the 24-h day, whereas external or endogenous effects can interfere with these rhythms. Although contact with their calves allows cows to behave more naturally, it is possible that calves demanding their mothers' attention may affect the cows' rhythmicity, similar to other external factors. To test this hypothesis, 2 herds of German Holstein cows housed in a mirrored loose housing system were included in the study, which was conducted over 2 experimental periods. Three treatments were applied, differing in contact between cow and calf. The contact dams had either whole-day or daytime contact with their calves, and the no-contact cows were separated from their calves directly postpartum. Accelerometers were used to record and analyze the cows' activity between 59 and 83 DIM, thus excluding the calving and weaning phases. Generalized linear mixed models were used to estimate the effect of treatment (no, daytime, and whole-day contact) on DFC and DI, considering the effects of estrus, deviation of milking start in the evening, and parity (primi- vs. multiparous). Finally, the harmonic period lengths of the activity patterns were extracted to analyze the distribution of the primarily expressed period lengths of the different treatments. In general, the average activity patterns of the cows did not differ between the treatments. However, dams with whole-day contact showed a lower activity peak before milking but a higher activity after evening milking. Nevertheless, the DFC and DI were similar in each group. During estrus, the chance of a maximum DFC decreased and the DI increased. Whole-day contact dams showed the most significant harmonic periods (33 per cow). Nevertheless, the primarily expressed period length (3.4 h) was equal in each treatment. In conclusion, neither contact with the calf nor its daily duration affected the ultradian and circadian rhythms of dams compared with cows separated from their calf.

## Highlights

• Whole-day or daytime contact with calves had no effect on rhythmicity of dams.
• Estrus highly influenced ultradian and circadian rhythms of cows.
• Cows primarily expressed rhythms of 3.4-hour period length.

## Graphical Abstract

**Figure:**
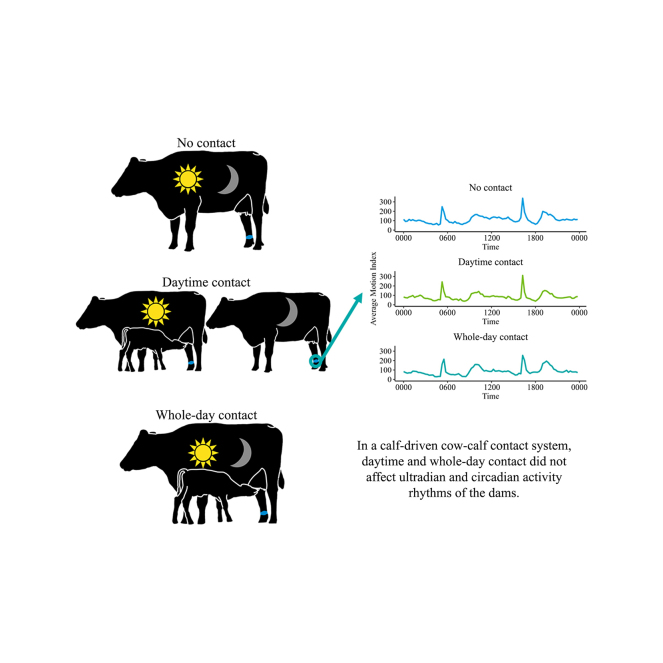
**Summary** This study investigated the effect of contact with their calf on dams' ultradian and circadian activity rhythms. The activity of cows with whole-day, daytime, and no contact with their calves was recorded. This activity was analyzed by comparing the average daily activity pattern to identify group differences. Additionally, the ultradian and circadian rhythms were analyzed by using the degree of functional coupling and the diurnality index. The results showed that the activity pattern of cows was similar for each group, and neither whole-day nor daytime contact with their calf affected the dams' ultradian or circadian rhythms.

The interest of consumers and farmers in prolonged cow-calf contact is increasing (Agenäs, 2020), and several dairy farms have already implemented cow-calf contact (**CCC**) systems in many variants. In this context, contact time during the day is a distinguishing characteristic (Eriksson et al., 2022) that may also have an impact on the dams. Under semi-natural conditions, the main suckling times of *Bos indicus* are at dawn and in the late afternoon (Reinhardt and Reinhardt, 1981). Holstein dairy cows in a cow-driven CCC (see definitions by Sirovnik et al., 2020) also mainly attempted to visit their calves during daytime, with the highest occurrence between 1800 and 2159 h (Johnsen et al., 2021). However, Roadknight et al. (2022) found that cows with only nighttime contact showed more agonistic behavior when re-united with their calf than cows that were in contact with their calf during the whole day (**WDC**), suggesting a negative impact of the shortened contact duration.

Although dams are highly motivated to visit and interact with their calves (Wenker et al., 2020), lying time and activity per day are not affected by WDC or part-time contact (Johnsen et al., 2021; McPherson et al., 2022). Nevertheless, activity patterns, and therefore circadian and ultradian rhythms, can be affected by endogenous and external factors such as calving, estrus, disease, social distress, and other external stressors (Berger et al., 2003; Wagner et al., 2021). Scheibe et al. (1999) and Berger et al. (2003) have shown that the degree of functional coupling (**DFC**) can be used to study the circadian rhythm of animals, based on their activity patterns. The DFC expresses the extent to which the measured activity is cyclic to 24 h, and therefore harmonically synchronized with the periodicity of the environment. Its specialty is to apply harmonic periods. Periods are defined as harmonic by dividing 24 h by an integer, resulting in 24 h, 12 h, 8 h, and so on (Fuchs et al., 2022). Until today only a pilot study (14 cow-calf pairs) using spectral entropy investigated the effect of CCC on the cows' circadian rhythm, and did not find significant differences between WDC, nighttime contact, and no contact (McPherson et al., 2022). In view of these results and the fact that the contact with the calves corresponds to natural conditions, one might expect a high degree of adaptation on the part of the dams. Nevertheless, it is possible that particularly calf-driven CCC, where the calf decides on the time point and duration of the contact, affects the dam's rhythmicity, similar to other external factors. Additionally, the sample size of the pilot study was small, and the stressful periods of the final separation of cow and calf were included. Furthermore, ultradian rhythm and daytime contact (**DTC**) were not considered. Therefore, we investigated the effect of DTC and WDC as well as no contact (**NOC**) with their calves on ultradian and circadian rhythms of cows.

The experiment was conducted on the research farm of the Thünen Institute of Organic Farming in Northern Germany and was split in 2 experimental periods (August 2020–April 2021 and August 2021–June 2022). The local Animal Welfare Committee was consulted beforehand, and because commercially available sensors were used and the cows were kept in their normal living conditions with no procedures that deviated from standard husbandry in CCC systems, it was decided that no ethical approval was necessary.

Two herds of German Holstein cows (polled/horned) were held in one mirrored barn with low bed cubicles. The polled herd included on average 43 (32–47) and the horned herd 39 (28–46) animals, respectively. Each side of the barn consisted of a separate cow and calf area (description in Wagner et al., 2012). The calves in contact with their dams could enter the cows' resting area using an automated gate (see Johnsen et al., 2016). Farm management followed the Council Regulation of organic farming (EU-VO 2018/848; Council Regulation of European Commission, 2018).

All cows were milked twice daily starting around 0500 and 1600 h (CET or CEST) in a tandem parlor. Fresh feed was provided at the feeding table during milking so that cows had access to fresh TMR after milking. The feed was additionally pushed 6 times a day on average. New bedding (straw) was dispensed into the cubicles twice per week.

Three treatments were applied: each herd contained a group of dams with contact with their own calves (contact group) and NOC cows (control) that were separated from their calves shortly after calving. During the first experimental period, the polled herd included dams that had WDC with their calves, and the horned herd included dams that had DTC. In the following period the contact time was changed; thus, the horned dams had WDC and the polled dams DTC. The WDC calves could enter the cows' area any time except during milking times, and DTC calves could enter the cows' area between the morning and the evening milking. This meant that the contact calves were always able to suckle when their mothers were also present in the cows' resting area. Cows and calves were randomly allocated to the contact or NOC groups directly after calving, stratified by calf sex and parity (primi- and multiparous). The contact dams and calves stayed in the maternity pen for 5 ± 1 d to strengthen their bond. Afterward, they returned to their herd and the calves were trained to use the automated gate starting a calf-driven system. The NOC cows were returned to the herd 2 ± 1 d after calving, and their calves were reared artificially. All calves were fed with milk for at least 90 d. The contact calves received milk from their dams by suckling and the control calves were fed from an automatic feeder. One cow had twins, with one calf being artificially reared and the other suckled (cow classified as WDC dam).

All management times (e.g., start and end of milking or time of feeding) as well as management events, such as bedding or claw trimming, were recorded. Cow-related data (e.g., the day of calving, the day of estrus, or day of health issue) were collected by farm staff or the management program.

The activity of each cow was recorded using 3-axis accelerometers (IceTags 3D) attached to the right hind leg of the cows. To exclude the influence of calving and weaning, and due to supply shortage of sensors in experimental period 2, the analysis of the activity data referred to the period of 59 to 83 DIM. Number of steps and motion index (**MI**) for each minute was calculated using IceTag Analyzer 2010 Version 4.005. Further data management and statistical analyses were performed using R Version 4.3.1 (R Core Team, 2023). Unreliable data due to sensor issues were excluded; if either step or MI was recorded as 0 for more than 12 h, the complete day of that cow dataset was excluded.

The DFC and diurnality index (**DI**) were calculated using R package digiRhythm (Nasser et al., 2023). The DFC can take on a value between 0 and 1, where 1 indicates a complete adaptation to the external 24-h day. The calculation of the DFC is based on the approach of Sinz and Scheibe (1976). However, within the digiRhythm package, the calculation of the different frequencies of activity bases on a Lomb-Scargle periodogram (Lomb, 1976; Scargle, 1982) instead of Fourier transformation used by Sinz and Scheibe (1976). Subsequently, significant frequencies were identified using the Baluev method (Baluev, 2008) with a significance level of *P* ≤ 0.05. The DFC was calculated using a sliding 7-d window.

The DI was calculated according to Hoogenboom et al. (1984) and shows diurnal and nocturnal activity, where 1 indicates complete diurnal activity and −1 indicates complete nocturnal activity. We defined day as the time between morning and evening milking (approximately 7 h) and night between evening and morning milking (approximately 10 h). A sliding DI was used because of Daylight Saving Time changes. For this purpose, instead of using the mean of milking start and end over the whole experimental period, the sliding mean of milking start and end over 7 consecutive days was used to define day and night. Before calculating the DFC and DI, the activity data as well as the management data were converted from CET and CEST to GMT. In addition, the first, last, and incomplete days were excluded from each dataset. Finally, the data were sampled at a 15-min interval, by summation of the MI of each minute.

All management times were checked for validity. Because the milking times were maintained after the Daylight Saving Time changes and an adaptation of the cows to the long-term deviation of milking time was observed, the short-term deviation rather than the milking time itself was used for the analysis. Therefore, the mean of the milking start of the day in question and the following 6 d was calculated as baseline. Subsequently, the difference in minutes between this mean and the milking start of the considered day was calculated.

To analyze the activity patterns, the average MI of all cows per treatment was plotted on a line graph at 15-min intervals per day (one line graph per treatment). In addition, plots for estrus and diestrus, horned and polled cows, primi- and multiparous cows, and each week in milk were created to visually analyze their effects on cow activity patterns according to treatment.

To analyze the influence of the contact times, generalized linear mixed models were calculated using the glmmTMB package (Brooks et al., 2017). Correlating predictors were not included in the same model. Because the data were autocorrelated over the days, covariance structure autoregressive order-1 was used. Due to repeated measurements of some cows, the lactation number nested in cow nested in herd was used as a random effect. The dredge function of the package MuMIn (Bartoń, 2023) was used to find the best model according to the corrected Akaike information criterion. Contact time and estrus were included as fixed effects due to the hypothesis and high biological relevance. Effects of horn status and season were also tested but excluded due to lack of significance. The assumptions of the best models were tested using the DHARMa package (Hartig, 2022). Subsequently, the model results were analyzed using the emmeans package (Lenth, 2023). A post hoc power analysis was calculated setting α = 5%.

If no significant rhythm is expressed an invalid division by zero may occur when calculating the DFC. This was the case for 15% of the data. As the expression of no significant rhythm in the biological context is similar to expressing no harmonic rhythm, these data points were set to DFC = 0. Due to the frequency of occurrence of DFC = 0 (26%) and DFC = 1 (56%), and as no previous study reported a threshold for high or low adaptation to the circadian rhythm, estimated by the DFC, we decided to use a median separated binomial distribution in our model, similar to Fuchs et al. (2022). Median of the DFC was 1; therefore, each DFC <1 was set to 0, which resulted in 56% data points for DFC = 1 and 44% data points for DFC = 0.

To analyze the DI data a Gaussian linear mixed model was used. Because outliers influenced the model results significantly, they were excluded using the 1.5 interquartile range method, based on Tukey (1977).

The Lomb-Scargle periodogram was used to analyze the primarily expressed harmonic period lengths. To compare the proportion of these period lengths between the treatments, the average number of the harmonic periods per cow and the proportion of each harmonic period length were calculated per group.

The experiment was designed with a total of 100 cows (period 1: 46, period 2: 54). Due to stillbirth or health issues of either cow or calf, 10 cows were excluded from the final dataset. Further, 11 cow datasets had to be excluded because they contained less than 15 d of activity data between 59 and 83 DIM. In total, 79 cow datasets (period 1: 39; period 2: 40; WDC: 18; DTC: 25; NOC: 36) were used for the analysis of average MI, the harmonic periods, and the DI model (1,763 observations), of which 16 cows were included in both periods. As the DFC model was a binomial model, 3 additional cow datasets (WDC: 2; DTC: 1) had to be excluded due to missing variation within a cluster (DFC = 1 on each day). The DFC model was thus calculated on 1,694 observations from 76 cow datasets of 62 cows.

The best DFC model contained the fixed effects contact time (NOC, DTC, and WDC), estrus (yes or no), and deviance of milking start in the evening (min). The variance of the clusters was 9.21 and the calculated R^2^ of this model was 0.18. However, due to the high number of cow datasets, the statistical power was high (0.98). For DI, the best model included the fixed effects contact time, estrus, and parity (primi- or multiparous). The cluster variance was 0.001, the R^2^ was 0.47, and the statistical power was 1.00.

The average MI plots were similar for each treatment. However, WDC dams showed a lower activity peak before milking and a higher peak after milking than the other groups. This could be explained by interaction with their calves, as WDC dams were the only ones who had contact beyond evening milking. This corresponds with the results of Reinhardt and Reinhardt (1981), who reported the highest suckling rate during that period for semi-free-ranging cow-calf pairs. Additionally, Johnsen et al. (2021) showed the highest visitation rate (23%) between 1800 and 2159 h in a cow-driven CCC system. However, in our study the estimated mean DI did not differ between the contact groups, compared with the NOC group (Table 1). As the time of highest visitation rate in Johnsen et al. (2021) is defined as nighttime in our definition of DI and the DI of the WDC dams was not lower than the DI of the NOC group, visitation of calves after evening milking did not seem to affect the rhythmicity of the cows. In addition, there was no difference in the chance of maximum DFC when the WDC and DTC groups were compared with the NOC group. Our outcomes of the DFC and DI model confirmed the findings of the pilot study by McPherson et al. (2022), who found no difference in circadian rhythms of cows with WDC or NOC.

**Table 1:** Results of the generalized linear mixed models of the degree of functional coupling (DFC, binomial model) and the diurnality index (DI, Gaussian model) to compare the effect of contact time (whole-day contact [WDC], daytime contact [DTC], and no contact [NOC])^1^

Predictor	DFC model			DI model			
	Odds ratio	95% CI	*P*-value	Estimate	SE	95% CI	*P*-value
Intercept	3.29	1.14 to 9.51	0.03	0.10	0.03	0.04 to 0.16	<0.01
DTC	0.83	0.15 to 4.56	0.95	0.02	0.02	−0.03 to 0.07	0.52
WDC	1.49	0.21 to 10.39	0.85	−0.03	0.02	−0.08 to 0.02	0.38
NOC	Referent			Referent			
Estrus	0.10	0.02 to 0.43	<0.01	0.12	0.03	0.06 to 0.17	<0.01
Diestrus	Referent			Referent			
Milking start evening	0.98	0.94 to 1.03	0.39				
Multiparous				−0.01	0.02	−0.04 to 0.03	0.70
Primiparous				Referent			

1 Milking start evening = deviation of the start of milking in the evening of its average over 7 consecutive days, given in minutes.

The estimated mean of DI was >0 for each treatment (*P* < 0.01), indicating a higher diurnal than nocturnal activity. Piccione et al. (2011) also reported higher diurnal activity in lactating dairy cows without calf contact. Additionally, when using our definitions of night and day, the diurnal visitation rate reported by Johnsen et al. (2021) was slightly higher than the nocturnal one (56% vs. 44%).

Roadknight et al. (2022) have already shown that a longer period of separation during the day causes stress in cows when they rejoin their hungry calves, triggering avoidance behavior toward their very young calves. However, in our study, the calves were at least 59 d old and in contact with their dams since birth. Therefore, synchronization of the dam and calf rhythms seems very likely. As the NOC cows were kept in the same herd as the DTC or WDC dams, we could ensure that management and housing factors affected each group in a similar way. However, transmission effects on the NOC cows due to the presence of calves in their herd cannot be excluded.

During estrus, the chance on a maximum DFC was 90% lower than that during diestrus, and the DI was higher during estrus than during diestrus. These findings coincide with the results of Wagner et al. (2021), who reported deviations from the cows' circadian rhythm during estrus. In contrast to Fuchs et al. (2022), who reported an influence of lactation number on DFC, we did not find an effect of parity on rhythmicity, measured by DI.

Duration of milking influences the time budget of dairy cows, and especially lying time but also feeding time decrease when milking times are prolonged (Gomez and Cook, 2010). The deviation of the start of evening milking was relevant for our model. The data showed that an increasing delay of the start of evening milking resulted in a decrease of the probability of a maximum DFC independent of treatment.

The analysis of the harmonic period lengths revealed a slight difference in the average number of harmonic periods per cow between the treatments (NOC: 28 periods/cow, DTC: 27 periods/cow, WDC: 33 periods/cow). The primarily expressed ultradian rhythm had a period length of 3.4 h in each group (Figure 1). The second most frequently expressed period length in DTC and WDC cows was 4.8 h, while this period length was the third most frequently expressed period length in NOC cows. These short period lengths of 3.4 and 4.8 h were primarily expressed, as the cows showed short periods of high activity and resting behavior lasting around 1.7 and 2.4 h, respectively. However, this behavior of multiple activity changes is consistent with the daily activity patterns of lactating dairy cows (Piccione et al., 2011). Additionally, the 24-h rhythm that Berger et al. (2003) considered the central rhythm came second, third, or fourth in our study. However, it should be emphasized that the ultradian rhythm with a period length of 12 h is more frequent in WDC cows than in the other groups. Fuchs et al. (2022) also described a primarily expressed rhythmicity of 12- or 24-h period lengths by dairy cows in an automated milking system.

**Figure 1:**
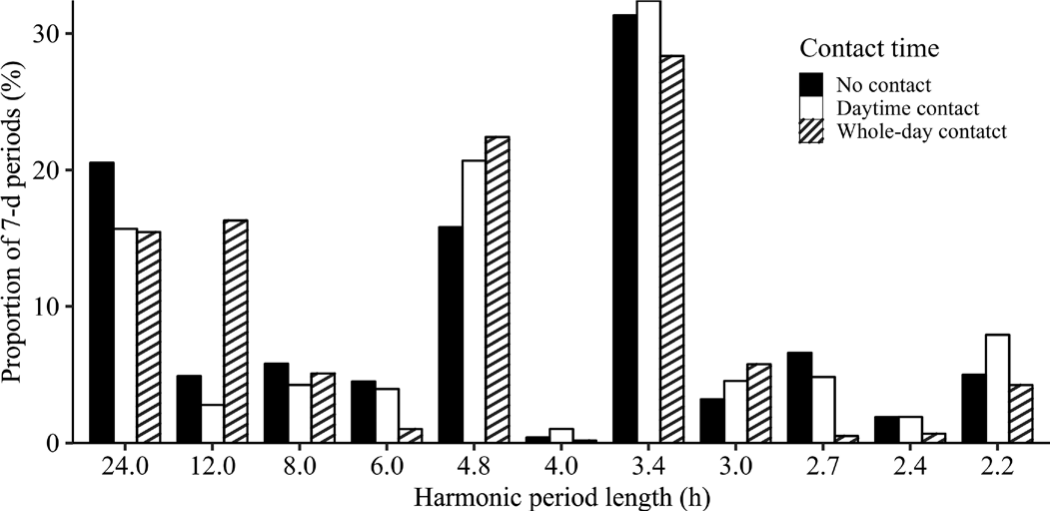
Proportion of the significant (*P* ≤ 0.05) harmonic 7-d periods per contact time. The no-contact cows (n = 36) expressed 999 harmonic periods; the daytime contact dams (n = 25) had 682 harmonic periods; and the whole-day contact dams (n = 18) expressed 589 harmonic periods.

In conclusion, whole-day contact with their calves slightly alters the activity of the cows but neither whole-day nor daytime contact affects their ultradian and circadian activity rhythm at the end of the early lactation. Therefore, calves do not interrupt the rhythmicity of their well-adapted dams, held under the presented conditions (freestall barn, milking parlor, calf-driven CCC). The effect of estrus was evident in our study and the effect of shifting the start of milking time seemed to be more important than expected. This should be tested in further studies.
